# Accelerated brain ageing in migraine: a multilevel MRI-based brain-age modelling study

**DOI:** 10.1093/braincomms/fcag110

**Published:** 2026-03-28

**Authors:** Hung-Yu Liu, Chen-Yuan Kuo, Pei-Lin Lee, Yi-Hsuan Liu, Wei-Ta Chen, Shih-Pin Chen, Yen-Feng Wang, Ching-Po Lin, Kun-Hsien Chou, Shuu-Jiun Wang

**Affiliations:** Department of Neurology, Neurological Institute, Taipei Veterans General Hospital, Taipei 112201, Taiwan; School of Medicine, National Yang Ming Chiao Tung University, Taipei 112304, Taiwan; Institute of Neuroscience, National Yang Ming Chiao Tung University, Taipei 112304, Taiwan; Center for Healthy Longevity and Aging Sciences, National Yang Ming Chiao Tung University, Taipei 112304, Taiwan; Institute of Neuroscience, National Yang Ming Chiao Tung University, Taipei 112304, Taiwan; Department of Neurology, Columbia University, New York 10032, USA; School of Medicine, National Yang Ming Chiao Tung University, Taipei 112304, Taiwan; Department of Neurology, Keelung Hospital, Ministry of Health and Welfare, Keelung 20148, Taiwan; School of Medicine, National Yang Ming Chiao Tung University, Taipei 112304, Taiwan; Brain Research Center, National Yang Ming Chiao Tung University, Taipei 112304, Taiwan; Division of Translational Research, Department of Medical Research, Taipei Veterans General Hospital, Taipei 112201, Taiwan; Department of Neurology, Neurological Institute, Taipei Veterans General Hospital, Taipei 112201, Taiwan; School of Medicine, National Yang Ming Chiao Tung University, Taipei 112304, Taiwan; Institute of Neuroscience, National Yang Ming Chiao Tung University, Taipei 112304, Taiwan; Brain Research Center, National Yang Ming Chiao Tung University, Taipei 112304, Taiwan; Department of Education and Research, Taipei City Hospital, Taipei 24213, Taiwan; Institute of Neuroscience, National Yang Ming Chiao Tung University, Taipei 112304, Taiwan; Brain Research Center, National Yang Ming Chiao Tung University, Taipei 112304, Taiwan; Department of Neurology, Neurological Institute, Taipei Veterans General Hospital, Taipei 112201, Taiwan; School of Medicine, National Yang Ming Chiao Tung University, Taipei 112304, Taiwan; Brain Research Center, National Yang Ming Chiao Tung University, Taipei 112304, Taiwan

**Keywords:** migraine, spatial distribution, MRI, brain age

## Abstract

Migraine is increasingly recognized as more than just an episodic headache disorder—it often carries a profound burden marked by emotional and cognitive disturbances. Recent insights point towards a possible link between migraine and accelerated brain ageing, yet the specific brain regions affected and the clinical implications remain poorly understood. We investigated grey matter-based brain age using voxel-based morphometry in 110 preventive treatment–naïve patients with migraine and 70 healthy controls, based on T1-weighted MRI and a validated brain-age prediction framework trained on data from 1318 healthy individuals. Compared with controls, patients with migraine exhibited a significantly higher global brain-age gap [mean difference = 4.24 years; confidence interval (CI, 0.12, 8.36); *P* = 0.039; η_p_² = 0.024]. Regionally, 66 out of 442 brain parcels—primarily within the prefrontal, frontal, cingulate, parietal and temporal cortices and the amygdala—showed elevated ageing patterns. Permutation-based canonical correlation analysis revealed a stable multivariate association between regional brain-age deviations and a combination of migraine-related clinical factors (headache frequency, painkiller usage and depressive symptoms), although individual factor contributions were variable. Functional annotation of the affected regions highlighted their roles in cognitive, emotional and perceptual processing. Together, these findings reveal that migraine is linked to widespread, regionally patterned brain ageing, mirroring the clinical and neurobiological complexity of the disease. This work not only deepens our understanding of migraine’s impact on the brain but also opens new avenues for exploring how timely intervention might protect brain health in this population.

## Teaser

Liu *et al.* report that patients with migraine show accelerated brain ageing compared with healthy individuals. Regionally, the prefrontal, frontal, cingulate, parietal, temporal and limbic areas are involved. These age-related changes relate to combined effects of headache frequency, painkiller use and depressive symptoms, underscoring migraine’s broad impact on brain health.

## Graphical Abstract

**Graphical Abstract:**
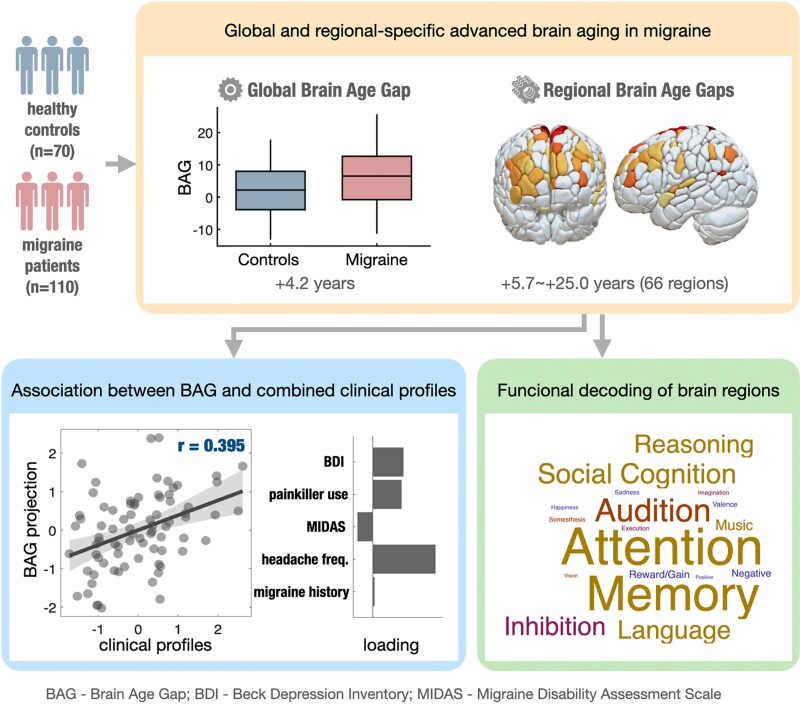

## Introduction

Migraine is a prevalent neurological disorder characterized by recurrent, debilitating headaches accompanied by troublesome symptoms.^1,2^ It significantly impacts various aspects of a person’s life. Beyond the headache itself, migraine affects emotional and mental health, especially in individuals with chronic migraine (CM; headache on ≥15 days/month for >3 months, with ≥8 days/month having migrainous features).^3^ Additionally, subjective cognitive impairment is a common complaint among patients with migraine.^4^ Clinic-based studies have reported poorer cognitive performance in migraineurs compared to healthy controls (HC).^5^ Moreover, recent studies suggest an association between migraine and an increased risk of dementia.^6^ These findings highlight the potential impact of migraine on brain health and motivate investigations into structural and functional brain changes, including alterations in brain ageing.

Brain age is a concept derived from neuroimaging studies that estimate an individual’s brain age by comparing their brain features to those in a normative dataset. A higher brain age, relative to chronological age, suggests the brain is undergoing accelerated age-related changes. A recent study showed an increased whole-brain-age gap (BAG) in patients with CM, while no such change was observed in those with episodic migraine (EM) compared to healthy participants.^7^ However, it remains unclear whether the increased BAG in migraine reflects global brain changes or region-specific alterations, and whether brain ageing is associated with clinical symptoms in patients with migraine.

The current study investigated brain age using grey matter-based MRI measures in patients with migraine who consecutively visited our headache clinic and compared the results with those of HC. Using voxel-based morphometry (VBM) to extract grey matter measures, we analysed the BAG both globally and across different brain regions, examined its relationship with migraine-related clinical profiles and explored the functional correlates of ageing brain regions.

## Materials and methods

### Patient consent

All participants completed informed consent forms after receiving a complete explanation of the study. The Institutional Review Board of our hospital approved the study protocol (2015-10-001BC; 2020-11-004C; YM108044F).

### Study subjects

Subjects aged 20–60 years with preventive treatment–naïve migraine, defined as those who had not received migraine preventive medications for at least 3 months prior to their visits, were prospectively evaluated by headache specialists at the Headache Clinic of Taipei Veterans General Hospital between 2015 and 2021. Patients with a history of major psychiatric, neurologic or systemic disease were excluded. Volunteers without a history of migraine, neurological or psychiatric disorders or major systemic diseases were recruited as HC. The entire study protocol was approved by the Institutional Review Board of the same hospital.

All participants completed a questionnaire during their first visit to provide demographic information. Patients with migraine also completed a semi-structured questionnaire to report their headache profiles and assessed their depressive symptoms using the Beck Depression Inventory (BDI).^8^ Headache frequency was defined as the average number of headache days per month over the past 3 months. Painkiller use frequency was defined as the average number of days per month that a patient took abortive painkillers during the same 3-month period. The duration of migraine history was defined as the number of years from when the patient first recalled experiencing migraines to the time of the current consultation. Functional disability caused by migraine in the past 3 months was assessed using the Migraine Disability Assessment Scale (MIDAS).^9,10^ The subtypes of migraine, including EM, CM and CM with medication overuse, were diagnosed according to the ICHD-3 criteria^3^.

### Image acquisition

All neuroanatomical T1-weighted images of the study subjects were acquired at Taipei Veterans General Hospital using a single 3.0 Tesla GE Discovery MR750 system (General Electric Healthcare, Milwaukee, WI, USA) equipped with an eight-channel phased-array head coil. To increase the available sample size, we incorporated T1-weighted scans acquired using two distinct acquisition protocols: (i) inversion recovery-prepared fast spoiled gradient-recalled echo (IR-FSPGR) and (ii) IR-FSPGR brain volume imaging (BRAVO) sequences. The acquisition parameters were optimized as follows with whole-brain coverage: repetition time = 9.4 ms (IR-FSPGR) or 9.2 ms (IR-FSPGR BRAVO); echo time = 4.0 ms (IR-FSPGR) or 3.7 ms (IR-FSPGR BRAVO); inversion time = 450 ms; flip angle = 12°; acquisition matrix = 256 × 256; field of view = 256 × 256 mm^2^; and number of excitations = 1. Volumetric data were acquired as contiguous axial slices (172 for IR-FSPGR; 168 for IR-FSPGR BRAVO) with 1 mm thickness, without gap or interpolation.

### Analytical flow of the study

**Figure 1:**
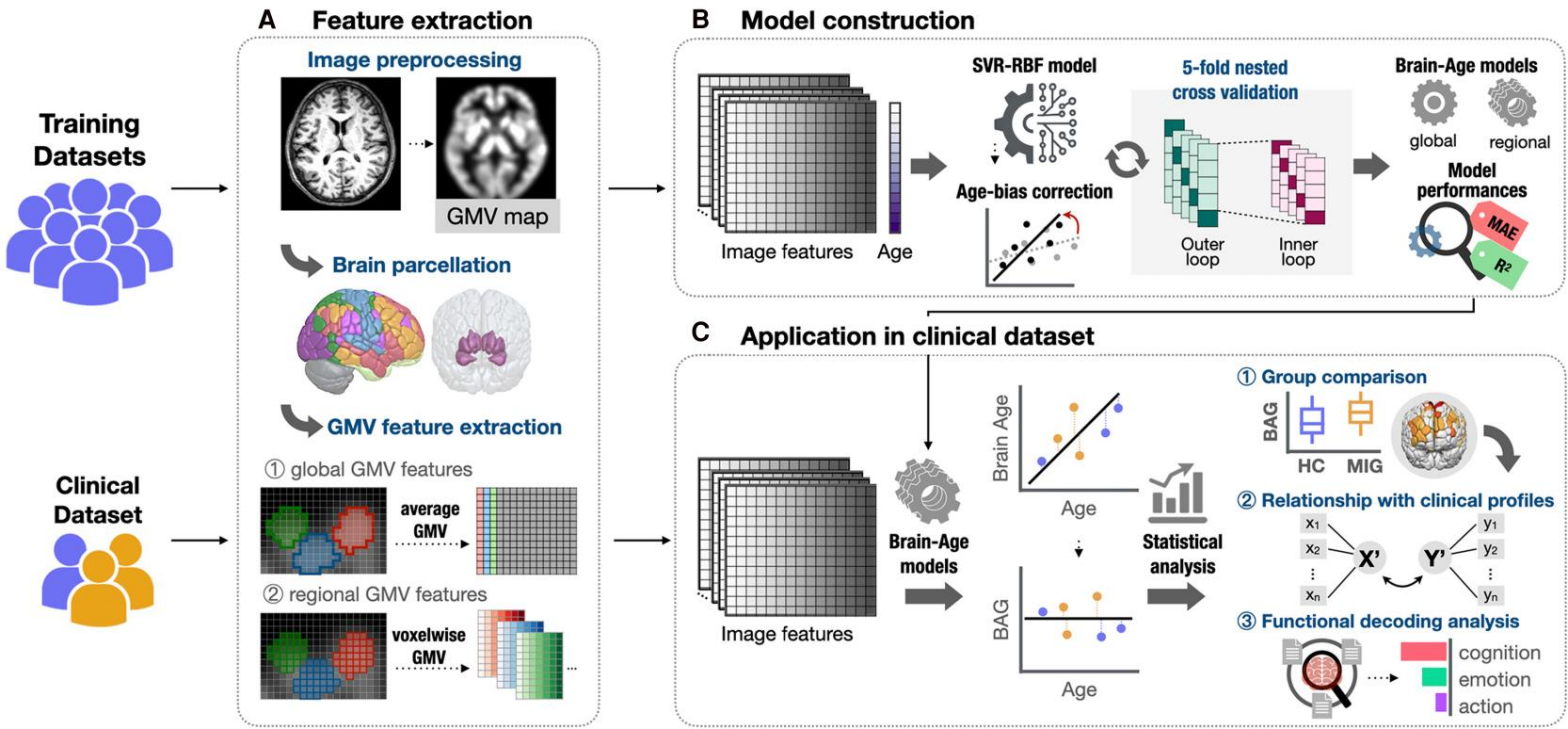
**Analytic flow of the study**. (**A**) Structural MRI data were processed using the VBM preprocessing pipeline, and voxel-wise and region-wise GMV features were extracted. (**B**) These imaging features were used to construct and validate global and regional brain-age predictive models in the training dataset. Model performance was evaluated, and the final brain-age predictive model was constructed for further application. (**C**) The established models were applied to the migraine cohort and HC to estimate individual brain ages, from which BAGs were calculated. Statistical analyses were conducted to compare global and regional BAG differences between groups. Additionally, associations between clinical profiles and BAG in migraine patients were examined. Finally, functional decoding analysis was performed to explore the brain functions and behavioural implications of migraine-associated regional BAG patterns. BAG = brain-age gap; GMV = grey matter volume; MAE = mean absolute error; MRI = magnetic resonance imaging; R^2^ = coefficient of determination; ROI = region of interest; SVR-RBF = support vector regression with radial basis function kernel; VBM = voxel-based morphometry.

The study framework includes the following steps: anatomical image preprocessing, feature extraction and brain-age model development using an independent normative population dataset and subsequent model application to the clinical migraine cohort and HC (Fig. 1).

### Image processing and feature extraction

Before initiating the image preprocessing protocols, comprehensive quality assurance was performed through visual inspection of all T1-weighted acquisitions to ensure optimal image quality and exclude potential neurological abnormalities. All neuroimaging datasets satisfied predefined quality criteria, specifically the absence of major motion or reconstruction artefacts.

Quantification of both voxel-wise and regional-wise grey matter volume (GMV) metrics from T1-weighted anatomical scans was accomplished through VBM analysis implemented in Statistical Parametric Mapping 12 (version 7771; Wellcome Institute of Neurology, University College London, UK) within the MATLAB computational environment (version R2021a; MathWorks, Natick, MA). The image preprocessing procedure and feature extraction protocol, building upon our previously validated GMV-based brain-age methodology,^11-13^ comprised the following sequential steps: (i) tissue class segmentation; (ii) generation of study-specific templates with subsequent spatial normalization to Montreal Neurological Institute (MNI) standard space utilizing the Diffeomorphic Anatomical Registration Through Exponentiated Lie Algebra algorithm^14^; (iii) computation of individual voxel-wise GMV maps through tissue modulation; (iv) smoothed with a 6 mm full width at half maximum Gaussian kernel; and (v) application of an explicit grey matter mask derived from averaged MNI-space probability maps (threshold: 0.2) to exclude low-probability grey matter voxels. Following the generation of individual GMV maps, we implemented a multi-atlas parcellation approach incorporating 400 Schaefer’s functionally defined cortical regions,^15^ 14 Harvard-Oxford subcortical structures^16^ and 28 regions from the spatially unbiased infratentorial cerebellar template.^17^ This comprehensive parcellation approach yielded 442 distinct brain regions, enabling the development of multi-level GMV-based brain-age prediction models which were in line with the recent implementation.^13^ Then, we extracted two complementary feature sets: mean GMV and voxel-wise GMV signatures within each region of interest (ROI). The voxel-wise GMV metrics were utilized for regional brain-age model construction, while mean volumes across all 442 regions were employed in developing the global brain-age model.

### Training dataset for developing brain-age models

The training cohort comprised T1-weighted MRI data collected through an in-house multi-site initiative (detailed cohort characteristics presented in Supplementary Table 1). To establish ethnically specific and methodologically rigorous brain-age prediction models at both global and regional levels, we consolidated neuroimaging data from 1318 neurologically healthy participants (687 females, 631 males; age range, 20–92 years; mean age ± standard deviation, 50.0 ± 19.3 years) across five Taiwanese imaging centres using six distinct MRI platforms. Site-specific acquisition protocols were optimized according to individual scanner configurations, with comprehensive imaging parameters and technical specifications provided in Supplementary Table 1. Those who had traumatic brain injury, structural brain abnormalities, major systemic conditions (including malignancies, cardiovascular events, respiratory insufficiency and end-stage renal disease) and major neurological or psychiatric disorders were excluded. All data acquisition procedures adhered to protocols approved by the respective institutional ethics committees, with documented informed consent obtained from all participants or their authorized representatives prior to study participation.

### Development and validation of brain-age prediction models

Support vector regression (SVR) with radial basis function (RBF) kernel was implemented to construct both global- and region-specific brain-age prediction frameworks, utilizing multi-level GMV metrics as predictive features for chronological age estimation. SVR-RBF has been widely adopted in brain-age prediction studies, given its capability to model non-linear relationships between brain characteristics and chronological age, while handling high-dimensional neuroimaging features and maintaining robust generalizability across diverse datasets.^18^ Model construction and validation were executed through the scikit-learn framework (version 0.23.2; https://scikit-learn.org/stable/), implementing a nested 5-fold cross-validation architecture with equipartitioned stratified subsampling.^19^ Hyperparameter optimization was conducted within the inner cross-validation loop via GridSearchCV, exploring cost parameter C and kernel coefficient gamma across a logarithmic parameter space (0.001–1000) while maintaining default configurations for the remaining SVR-RBF parameters. Model performance evaluation, conducted in the outer cross-validation loop, employed mean absolute error (MAE) and adjusted coefficient of determination (R^2^) to assess the correspondence between predicted and chronological age, with metrics averaged across validation iterations. The finalized global- and region-specific brain-age prediction models were subsequently trained on the complete training dataset using 5-fold cross-validation before application to the clinical migraine cohort.

### Application of brain-age models in clinical dataset

The feature extraction pipeline, using MNI-space tissue templates from the training cohort, was applied to the clinical dataset to quantify global and regional GMV. Subsequently, the validated multi-level brain-age prediction models were implemented to compute global- and regional-level brain age for each participant. To address the inherent estimation bias in regression-based approaches—particularly the ‘regression towards the mean’ phenomenon, which manifests as age-dependent over- and under-estimation—we implemented a systematic correction procedure for brain-age predictions.^20,21^ Specifically, a linear regression model was fitted to predict brain age from chronological age within the training dataset (1318 healthy participants; age 20–92; six MRI platforms). The resulting regression parameters were then applied to adjust brain-age estimates in the migraine cohort and HC, yielding the corrected brain-age values. The BAG was calculated as the difference between corrected brain age and chronological age, with positive values indicating accelerated brain ageing.^22^ Correlations between corrected BAG and chronological age in our study participants are shown in Supplementary Fig. 1A–C.

### Analysis of group differences

We used single-factor two-level analysis of covariance (ANCOVA) to compare BAG between patients with migraine and HC, adjusting for potential confounding variables, including chronological age, quadratic age effects, sex, total intracranial volume and MRI acquisition protocols. The analysis of the global BAG, a unified measure derived from whole-brain GMV patterns, constituted a single statistical test of a pre-specified hypothesis. The subsequent regional-level analyses required correction for multiple comparisons, which were implemented using a false discovery rate (FDR) approach across all 442 comparisons (FDR-corrected *P* < 0.05). Effect size for these ANCOVA analyses was quantified using partial eta squared (η_p_^2^) statistics

### Validation for global brain-age gap findings derived from different MRI protocols

Among the 110 migraine patients, 23 (20.9%) underwent IR-FSPGR scanning (43.09 ± 11.24 years, 3 males/20 females) and 87 (79.1%) underwent BRAVO scanning (37.14 ± 9.43 years, 34 males/53 females). For the 70 HC, 23 (32.9%) were scanned with IR-FSPGR (42.30 ± 9.75 years, 4 males/19 females) and 47 (67.1%) with BRAVO (34.06 ± 7.89 years, 20 males/27 females). Two additional analyses were conducted to validate the findings of BAG difference between patients with migraine and HC. First, we applied the ComBat harmonization^23^ approach to remove protocol-related variance. Second, we performed protocol-stratified analyses and conducted the comparisons separately.

### Correlations between brain-age gap and clinical profiles in migraine

We used permutation-based canonical correlation analysis (PermCCA) to examine the relationship between clinical profiles and regional BAG patterns in participants with complete clinical profile data (*n* = 80).^24^ This analytical approach identifies the maximally correlated linear combinations—known as canonical variates (CVs)—between two sets of variables. The first set comprised clinical metrics, including headache frequency, painkiller use frequency, scores of MIDAS and BDI and the duration of migraine history. The second set encompassed significant regional BAG alterations in patients with migraine. Given the extensive number of regions showing BAG differences (66 regions), dimensional reduction was implemented through principal component analysis, retaining components accounting for 50% of the variance of BAG alterations. Chronological age and sex were controlled as confounding effects for clinical profiles, while chronological age, quadratic age effects, sex, total intracranial volume and MRI acquisition protocols were controlled for neuroimaging measures. Quadratic age terms were included for neuroimaging measures to account for non-linear brain ageing trajectories that may persist after bias correction.^25^ Statistical inference was conducted through non-parametric permutation testing (10 000 iterations). The implementation code for PermCCA analysis is publicly accessible at https://github.com/andersonwinkler/PermCCA. To assess the stability of CCA results, we performed bootstrap resampling with 10 000 iterations, computing 95% confidence intervals for the canonical correlation and individual loadings. We further correlated the CV identified from clinical profiles with the 66 brain regions with altered BAG to determine the contribution of each regional BAG.

### Decoding functional roles of altered brain-age gap regions in migraine

We explored brain functions and behavioural implications of migraine-associated regional BAG patterns by using the Behavioral Analysis Plugin (version 4.0, May 2023) within Mango 4.1 (http://ric.uthscsa.edu/mango/).^26^ Significant regional BAG alterations were converted to binary masks and spatially normalized to Talairach space for analysis. This analytical framework maps the identified ROIs to functional annotations from the BrainMap database (http://brainmap.org),^27^ systematically assessing associations across five primary functional domains of brain (action, cognition, emotion, interoception and perception) and their 59 constituent sub-domains. The Behavioral Analysis Plugin calculates the spatial probability distribution by comparing the proportion of activation foci for each behavioural domain found within our identified regions against the proportion of that same behavioural domain across the entire BrainMap database. This procedure tests the null hypothesis that the distribution of activation foci observed within our ROIs does not differ from a spatially uniform random distribution across the brain. The resulting *Z*-scores quantify the statistical likelihood of functional associations, where *Z* > 3.0 represents significant association after Bonferroni correction for multiple comparisons across 59 behavioural sub-domains (*P* < 0.05).

### Statistical analysis of clinical data

The distribution of normality of continuous variables was tested using the Kolmogorov–Smirnov test. Variables that are distributed normally (*P* > 0.05; age, duration of migraine history, grey matter, white matter and total intracranial volumes) were presented as means ± standard deviations, and Student’s *t*-test was used when compared these variables between groups. Variables that are not distributed normally (*P* < 0.05; frequency of headache and migraine, frequency of painkiller use, scores of MIDAS and BDI and cerebrospinal fluid volume) were presented as median with interquartile range (IQR), and the Mann–Whitney U-test was used when comparing these variables between groups. The chi-squared test was used to test for differences in categorical data (i.e. gender). Two-tailed *P* < 0.05 were considered statistically significant in all analyses.

## Results

### Demographics and clinical profiles of migraine cohort and healthy control

The study included 180 participants: 110 patients with migraine (38.4 ± 10.1 years old, male/female: 37/73) and 70 HC (36.8 ± 9.3 years old, male/female: 24/46). There were no significant differences in age or sex distribution between the two groups. Patients with migraine had a median of 12 monthly headache days (IQR 5–20), 6 monthly migraine days (IQR 3–10), 5 monthly days of painkiller use (IQR 1–7), a MIDAS score of 18 (IQR 5.25–43.75) and a BDI score of 11 (IQR 6–17) (Table 1). Among patients with migraine, 71 patients had EM, 39 patients had CM and 21 patients had migraine with aura (6 with CM and 15 with EM). Patients with CM had a marginally higher BDI score than those with EM (15.1 ± 9.9 versus 11.1 ± 10.4, *P* = 0.066). Most patients had previously used acetaminophen or nonsteroidal anti-inflammatory drugs, with or without caffeine, as acute painkillers, and there was no substantial difference in painkiller use between patients with EM and those with CM. No patients had received calcitonin gene-related peptide monoclonal antibodies or gepants prior to the study. Among patients with CM, 12 patients had comorbid medication overuse. Patients with CM with or without medication overuse did not differ in headache frequency (*P* = 0.098), migraine frequency (*P* = 0.964), BDI (*P* = 0.971) or MIDAS (*P* = 0.835).

**Table 1:** Clinical and neuroimaging profiles of patients with migraine and HC

	Migraine (*n* = 110)	HC (*n* = 70)	*P* value
Age, years	38.4 ± 10.1	36.8 ± 9.3	0.283
Female, %	66.4%	65.7%	0.929
Migraine history, years	15.9 ± 10.3		
Headache days per month (IQR)	12 (5–20)		
Migraine days per month (IQR)	6 (3–10)		
Painkiller use, days per month (IQR)	5 (1–7)		
MIDAS (IQR)	18 (5.25–43.75)		
BDI (IQR)	11 (6–17)		
Total intracranial volume, mL	1407.1 ± 121.4	1459.0 ± 126.3	0.007
Grey matter volume, mL	705.9 ± 64.3	741.4 ± 61.0	<0.001
White matter volume, mL	396.8 ± 39.8	419.1 ± 40.1	< 0.001
CSF volume, mL	297.0 (258.0–334.0)	294.5 (261.8–335.3)	0.945

Student’s *t*-test was used for age, duration of migraine history and brain volume measures (except for CSF); the Mann–Whitney U-test for headache, migraine and painkiller use frequency, MIDAS and BDI scores and CSF volume; and the chi-square test for gender.

HC = healthy controls; IQR = interquartile range; MIDAS = Migraine Disability Assessment Scale; BDI = Beck Depression Inventory; CSF = cerebrospinal fluid; mL = millilitre.

Compared to HC, patients with migraine had a smaller total intracranial volume (*P* = 0.003), smaller GMV (*P* = 0.001), smaller white matter volume (*P* < 0.001) and similar cerebrospinal fluid volume (*P* = 0.837) after controlling for age, sex and MRI acquisition protocol (Table 1).

### Performance of brain-age prediction model in the training dataset

**Figure 2:**
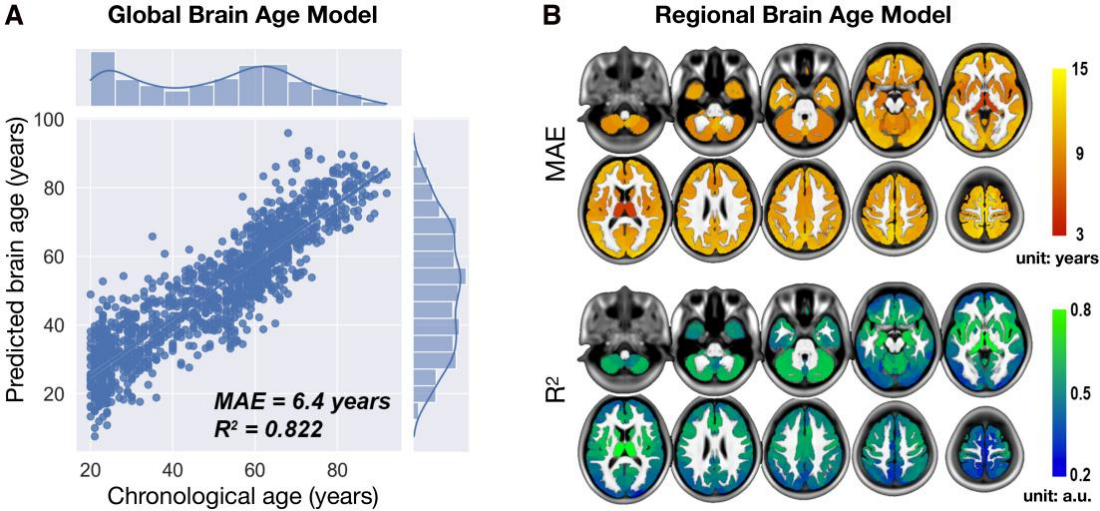
**Performances of global and regional brain-age prediction models in the training dataset**. (**A**) Global brain-age model: Pearson’s correlation analysis showed that global brain age was highly correlated with chronological age across the training dataset (*n* = 1318 healthy participants; SVR-RBF model, 5-fold nested cross-validation). Predictive accuracy was reflected by mean absolute error (MAE = 6.444 years) and coefficient of determination (R^2^ = 0.822). Each dot represents the predicted brain age of a single participant; histograms show the marginal distributions of chronological and predicted age. (**B**) Regional brain-age model: performance maps show voxel-wise MAE and R^2^ values, constructed using the same SVR-RBF model and training dataset (*n* = 1318), and demonstrate mild to good prediction (MAE range, 5.772–14.023 years; R^2^ range, 0.218–0.854). The top row shows regional MAE maps, with larger values indicate greater prediction error. The bottom row shows regional R^2^ maps, with higher values indicate better model fit. MAE = mean absolute error; R^2^ = coefficient of determination; a.u. = arbitrary units.

We demonstrated a significant relationship between chronological age and predicted global brain age in an independent training dataset. As anticipated, the predicted global brain age was strongly correlated with chronological age (MAE = 6.444; R^2^ = 0.822; *P* < 0.001). Additionally, the regional brain-age prediction model exhibited mild to good predictive performance, with MAE ranging from 5.772 to 14.023 years and R^2^ values ranging from 0.218 to 0.854 (Fig. 2A and B).

### Group differences in global brain-age gap

**Figure 3:**
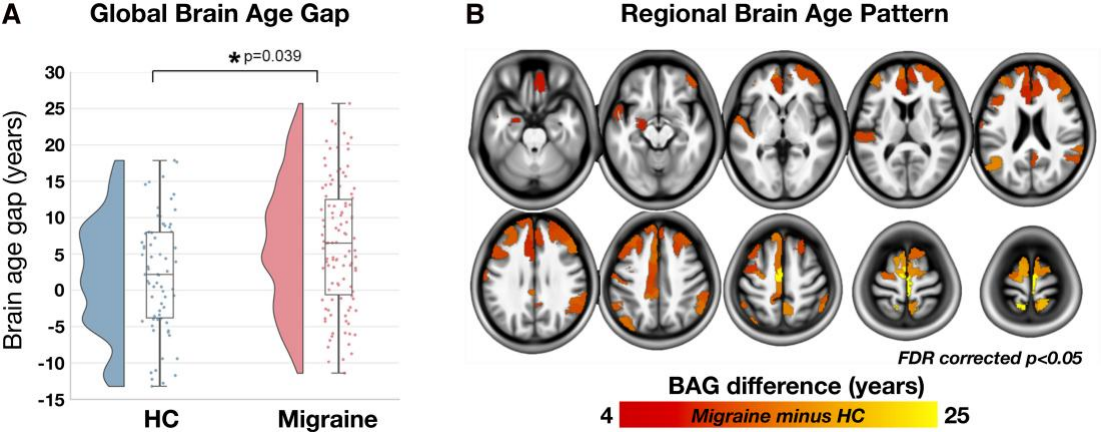
**Global BAG and regional BAG pattern in patients with migraine**. (**A**) Global BAG was significantly higher in patients with migraine compared to HC [mean difference = 4.24 years; *F*(1,173) = 4.313; *P* = 0.039; 95% CI (0.12, 8.36); η_p_^2^ = 0.024; *n* = 110 migraine patients; *n* = 70 HC]. Each dot represents the BAG value of a single participant; the violin plot on the left shows the data distribution, and the box plot on the right shows the median and IQR. (**B**) Regional BAG was increased in 66 out of 442 brain regions in patients with migraine (*n* = 110) compared to HC (*n* = 70), mainly in cortical regions (FDR-corrected *P* < 0.05). The colour bar indicates the magnitude of BAG difference (years). One-way ANCOVA, controlling for chronological age, quadratic age effects, sex, total intracranial volume and MRI acquisition protocols, was conducted for both analyses. BAG = brain-age gap; FDR = false discovery rate; HC = healthy controls.

After controlling for chronological age, the quadratic term of age, sex, total intracranial volume and MRI acquisition protocol, patients with migraine exhibited a significantly higher global BAG compared to HC [mean difference = 4.24 years; 95% CI (0.12, 8.36); *P* = 0.039; η_p_^2^ = 0.024; Fig. 3A].

In patients with migraine, global BAG was not significantly associated with headache frequency (*P* = 0.074), migraine frequency (*P* = 0.466), painkiller use frequency (*P* = 0.687), BDI scores (*P* = 0.412), MIDAS scores (*P* = 0.918) or duration of migraine history (*P* = 0.978). When comparing global BAG among patients with EM, CM and HC, we found significant differences across the three groups [*F*(2,172) = 4.376; *P* = 0.014; η_p_^2^ = 0.048]. *Post hoc* analyses showed that patients with CM had a higher global BAG compared with both HC [95% CI (1.46, 7.35); *P* = 0.004] and EM [95% CI (0.17, 6.19); *P* = 0.038]. In contrast, patients with EM did not differ from HC in global BAG [95% CI (−1.28, 3.73); *P* = 0.377]. Additionally, patients with CM and medication overuse did not show a different global BAG compared with patients without CM and medication overuse [mean difference = 2.56 years; 95% CI (−8.10, 5.33); *P* = 0.677; η_p_^2^ = 0.006].

### Validation of global brain-age gap findings

The ComBat harmonization approach revealed that migraine patients continued to demonstrate significantly higher global BAG compared to controls [mean difference = 3.93 years; 95% CI (0.82, 5.39); *P* = 0.008; η_p_^2^ = 0.04], providing robust validation of our original finding. Then, we stratified our patients by MRI protocol and found that the group differences in the two protocols showed consistent trend in the same direction as the combined analysis, respectively, although they did not reach statistical significance [IR-FSPGR: migraine: 0.315 ± 6.845 years; control: −4.115 ± 6.424 years; mean difference = 4.43 years; 95% CI (−0.56, 7.17); *P* = 0.092; η_p_^2^ = 0.069; BRAVO: migraine: 7.989 ± 8.528 years; control: 5.206 ± 6.457 years; mean difference = 2.78 years; 95% CI (−0.49, 5.06); *P* = 0.106; η_p_^2^ = 0.020].

### Group differences in regional brain-age gap

Sixty-six out of 442 brain regions showed increased BAGs in patients with migraine compared to HC (FDR-corrected *P* < 0.05). These regions were primarily located in cortical areas, including the dorsolateral and medial prefrontal cortex, orbitofrontal cortex, primary motor and somatosensory cortex, posterior parietal cortex, cingulate cortex and auditory and visual association cortices, as well as the subcortical limbic structure, the amygdala (Fig. 3B; Supplementary Table 2). No brain region showed a decreased BAG in patients with migraine.

### Relationship between regional brain-age gaps and clinical profiles in patients with migraine

**Figure 4:**
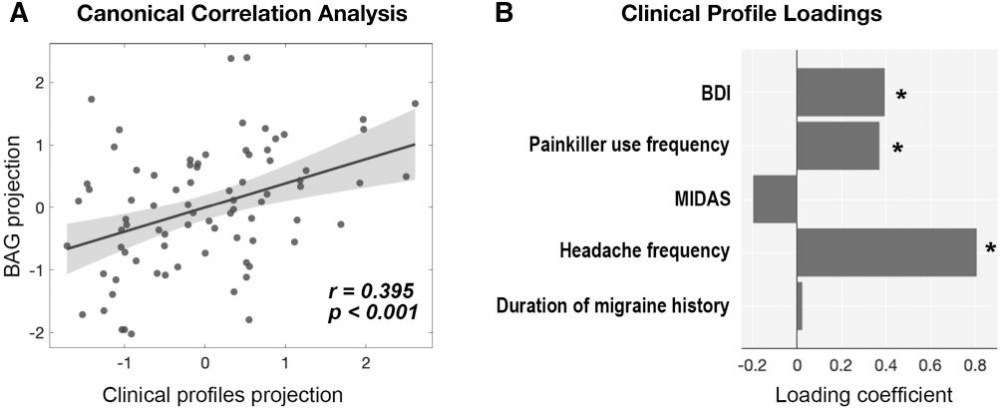
**Relationship between BAGs and clinical profiles in patients with migraine**. (**A**) Permutation-based canonical correlation analysis revealed a mild-to-moderate positive correlation between the CVs of clinical profiles and regional BAG alterations (*r* = 0.395; *P* < 0.001; *n* = 80). Each data point represents one patient’s CV projection; the shaded area denotes the 95% confidence interval of the regression line. (**B**) Standardized loadings of the clinical variables in the permutation-based canonical correlation analysis. An asterisk (*) indicates a significant association with BAG in the analysis (FWE-corrected *P* < 0.05; *n* = 80). Statistical significance was determined using non-parametric permutation testing (10 000 iterations). Only patients with complete clinical data were included for the above analyses. BAG = brain-age gap; BDI = Beck Depression Inventory; FWE = family-wise error; MIDAS = Migraine Disability Assessment Scale.

Regional BAG increases were not significantly associated with headache frequency, migraine frequency, painkiller use frequency, BDI or MIDAS scores or duration of migraine history. We applied PermCCA to examine the relationship between regional BAGs and clinical profiles in patients with migraine. The CVs of regional BAGs and clinical profiles showed a mild-to-moderate correlation (*r* = 0.395; *P* < 0.001; Fig. 4A). Regional BAGs were significantly associated with headache frequency (loading = 0.806; *P* < 0.001), painkiller use frequency (loading = 0.370; *P* < 0.001) and BDI scores (loading = 0.394; *P* < 0.001) (Fig. 4B). Bootstrap analysis showed that overall canonical correlation was significant and stable [*r* = 0.395; 95% CI (0.006, 0.626)], while individual canonical loadings varied across bootstrap samples: headache frequency [loading = 0.806; 95% CI (−0.386, 1.294)], painkiller use frequency [loading = 0.370; 95% CI (−0.923, 0.780)], BDI [loading = 0.394; 95% CI (−0.752, 0.951)], MIDAS [loading = −0.197; 95% CI (−1.114, 0.631)] and duration of migraine history [loading = 0.024, 95% CI (−1.236, 0.678)]. The brain regions significantly associated with the clinical profile CV are listed in Supplementary Table 3.

### Functional decoding of brain regions with altered brain-age gap

**Figure 5:**
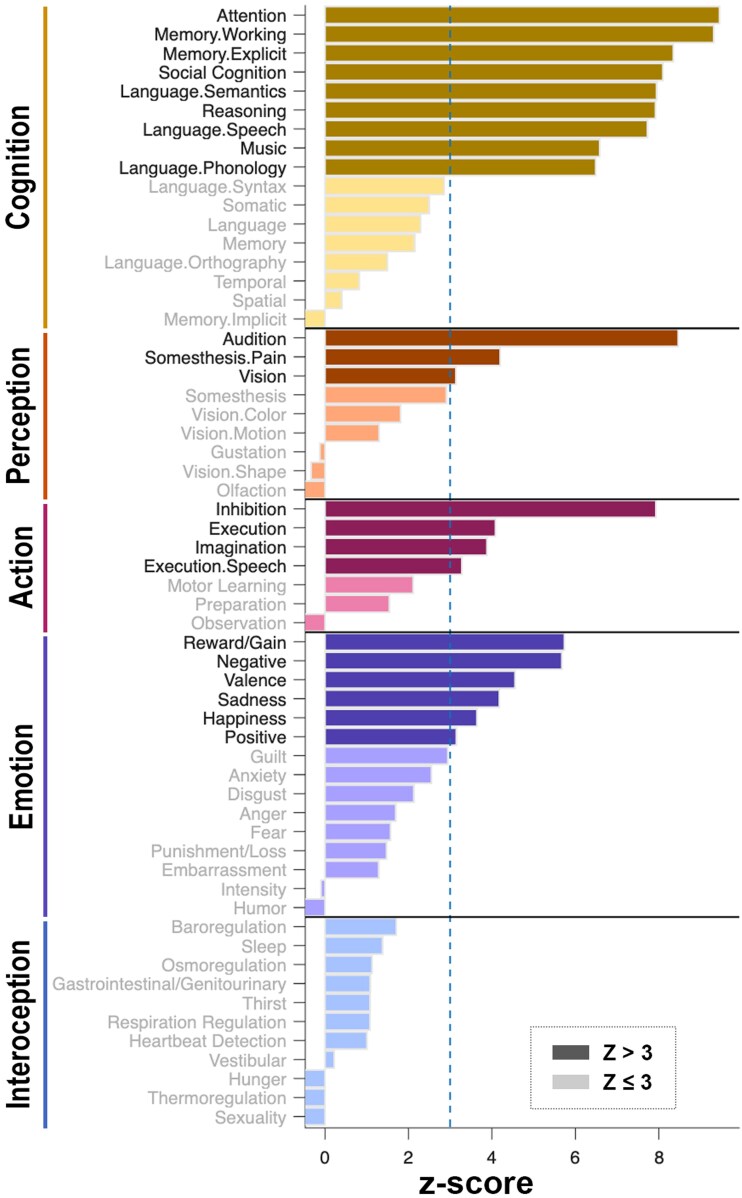
**Functional roles of altered BAG regions in migraine**. Meta-analytic functional decoding identified behavioural domains associated with brain regions showing increased BAG in patients with migraine (*n* = 110) compared to HC (*n* = 70). Bars indicate *Z*-scores of functional associations, derived from binomial distribution testing comparing observed activation probabilities within ROIs against expected probabilities under the null hypothesis of uniform spatial distribution. (**A**) Z-score greater than 3.0 (indicated by the vertical dashed line) represents a significant association after Bonferroni correction for multiple comparisons across 59 behavioural sub-domains (Bonferroni-corrected *P* < 0.05). Significant associations were observed in four of the five primary functional domains (cognition, perception, action and emotion), with cognition being the most strongly implicated. No brain regions showed significantly lower BAG in patients with migraine compared to HC. BAG = brain-age gap; ROI = region of interest.

Brain regions with increased BAG in patients with migraine were annotated in four out of five primary functional domains: cognition, perception, action and emotion. Among these, cognitive functions were the most prominently involved, including attention, working memory, social cognition, music cognition, reasoning and language. Additionally, regions with increased BAG were associated with auditory processing and inhibitory control (Fig. 5).

## Discussion

Our study demonstrated evidence of accelerated brain ageing in patients with migraine. The affected regions spanned a wide range of brain areas and functional domains and were associated with clinical features and comorbidities.

Previous neuroimaging studies in migraine have demonstrated structural and functional changes of brain that are involved in pain processing, sensory integration and cognitive functions compared to those in the healthy subjects.^28,29^ In this study, we applied a brain-age model to analyse brain volume changes in patients with migraine from the perspective of brain ageing. Consistent with a recent study demonstrating increased global BAG in patients with CM,^7^ our findings also revealed elevated BAG in patients with migraine, particularly those with CM, suggesting that increased migraine burden could relate to changes in brain health. Moreover, increased BAG was observed at both global and regional levels. Although the global BAG differed between patients with migraine and HC, the considerable overlap between their distributions suggests that the effect operates at the group level rather than distinguishing individuals. It is important to note that different brain regions age at varying rates and are influenced by multiple factors, including genetics, lifestyle and both internal and external environmental conditions.^30^ This regional variability in brain ageing aligns with our observation that the regional BAGs, rather than the global BAG, showed greater effect sizes, indicating region-specific vulnerability to migraine-related neuroplastic changes. The involved regions encompassed the prefrontal and frontal cortices, anterior cingulate gyrus, left temporal and bilateral parietal cortices and the amygdala. The affected regions largely overlap with networks involved in pain perception and modulation, including sensory–motor, affective–emotional and cognitive–control systems, as previously demonstrated in functional MRI studies. Structural changes in sensorimotor areas, such as the precentral and postcentral gyri, may reflect altered sensory integration and contribute to hypersensitivity to external stimuli commonly reported in migraine.^31^ In contrast, accelerated ageing in the frontal and cingulate cortices and the angular gyrus—key hubs for affective and cognitive pain regulation—could impair top-down inhibitory control, contributing to pain chronification and emotional dysregulation.^32^ Moreover, alterations in the temporal and limbic regions support the view of migraine as a disorder of the neurolimbic pain network and may underlie its frequent psychiatric comorbidities.^33^ Together, these findings suggest that region-specific patterns of brain ageing in migraine may reflect neuroplastic changes associated with the disorder.

We found that in patients with migraine, regional brain ageing patterns were associated with the combination effect of clinical factors, including headache frequency, painkiller use frequency and depression, although no significant association was observed when examining each clinical factor alone. This finding suggests that migraine-related brain-age acceleration likely reflects the cumulative burden of multiple clinical factors rather than the effect of any single variable. Indeed, during bootstrap analysis, the contribution of individual clinical variables appeared unstable, whereas their combined effects remained stable. The observed variability in the contribution of each clinical factor reflects the limited sample size relative to the model’s complexity, as well as the intrinsic clinical heterogeneity of migraine, in which patients present with diverse symptom profiles and comorbidities. MIDAS, however, was not associated with regional BAGs in this multivariate model. Unlike headache frequency and painkiller use that directly reflect headache exposure, MIDAS measures disability and can be influenced by non-biological factors, such as work flexibility or coping strategies. Therefore, MIDAS may not align linearly with biological ageing measures once frequency and psychological burden are accounted for. These findings suggest that migraine-related brain ageing and clinical manifestations could be interrelated through shared mechanisms. Ageing-related processes, such as impaired pain modulation, neuroinflammation and reduced neuroplasticity,^27,34^ may contribute to the persistence of pain states. Moreover, diminished executive control and altered decision-making observed in older adults may resemble mechanisms underlying medication overuse in CM.^35,36^ Thus, the observed associations between BAG, headache burden and analgesic use may reflect shared neurobiological mechanisms and potentially bidirectional influences.

We did not assess cognitive function in the participants of this study. Although functional decoding provides useful context by linking structurally vulnerable regions to meta-analytic functional associations, these associations do not demonstrate that the identified regions directly mediate those functions in patients with migraine. This reverse-inference caveat should be considered when interpreting the results. Nevertheless, the functional decoding maps suggest that the brain regions showing altered brain age are implicated in a wide range of cognitive, emotional and perceptual processes, providing a potential context for understanding the multifaceted impact of migraine on the brain. This finding echoes numerous clinic-based studies reporting that patients with migraine exhibit poorer performance across various cognitive domains compared to HC, such as executive function, language, attention, working memory, planning, visual spatial memory and visuomotor processing speed.^5,6^ This finding might also suggest the presence of psychiatric comorbidities commonly observed in patients with migraine.^37^ Taken together, our findings raise the possibility that migraine may be associated with accelerated brain ageing with neurobiological changes in brain regions implicated in cognitive, emotional and perceptual functions. These findings underscore the potential long-term impact of migraine on brain health. Whether the use of preventive medications can reverse brain ageing remains an important question for future research.^38^

Several limitations should be considered when interpreting our results. First, as this is a clinic-based study, the enrolled patients may have a higher clinical burden of migraine compared to those in the general population. Therefore, the findings may not be generalizable to community-based migraine patients. In addition, brain-age model performance can vary with ancestry, demographics and scanner ecosystems. As both the clinical cohort and normative model were Taiwanese, this may also limit generalizability. Second, due to the cross-sectional design, a causal relationship between increased BAG and migraine cannot be established. Third, cognitive function was not assessed in this study. Whether the observed accelerated brain ageing on MRI is associated with corresponding cognitive impairment remains to be determined and warrants further investigation. Fourth, the increase in global BAG in patients with migraine showed borderline significance, warranting cautious interpretation. Additionally, the use of two MRI protocols may have introduced variability despite statistical adjustment. Notably, ComBat harmonization of the two protocols strengthened the statistical significance. Although the stratified analyses were underpowered, both protocols showed consistent trends with the combined results. Fifth, we did not obtain BDI score in our HC, which may partially influence brain-age differences between groups.

To conclude, patients with migraine exhibited region-specific patterns of accelerated brain ageing. Future studies incorporating multimodal imaging features—including white matter integrity, diffusion metrics and other morphometric measures—along with a longitudinal design, may provide a more comprehensive understanding of brain ageing in migraine and help identify potential treatment strategies to mitigate these changes.

## Supplementary Material

fcag110_Supplementary_Data

## Data Availability

The data that support the findings of this study are available from the corresponding author upon reasonable request. The analyses in this study were performed using a combination of commercial and open-source software: commercial software, MATLAB (https://www.mathworks.com/); open-source software and toolboxes, SPM12 (https://www.fil.ion.ucl.ac.uk/spm/docs/); Python (https://www.python.org/); Scikit-learn library (https://scikit-learn.org/stable); PermCCA (https://github.com/andersonwinkler/PermCCA); Mango (https://mangoviewer.com/plugin_behavioralanalysis.html).
