# Supplementary material for: Accelerated brain ageing in migraine: a multilevel MRI-based brain-age modelling study
Source: Brain Commun. 2026 Mar 28;8(2):fcag110. doi: 10.1093/braincomms/fcag110 (PMC13056717; doi:10.1093/braincomms/fcag110)
Supplement: fcag110_Supplementary_Data [file fcag110_supplementary_data.pdf]

Supplementary Table 1. Data sources for brain age training cohort

**Cohort**	**N**	**Age** mean±SD [range]	**Sex** male/female	**Scanner**	**Coil**	**Sequence**	**Protocol parameters**
National Yang Ming Chiao Tung University	804	46.4±20.4 [20-92]	404/400	Siemens Tim Trio (3T)	12-channel / 32-channel phased-array head coil	MPRAGE	TR/ TE/ TI = 3500/3.5/ 1100 msec; flip angle = 7°; NEX = 1; FOV = 256 x 256 mm^2; matrix size = 256 x 256; 192 sagittal slices; and voxel size = 1.0 x 1.0 x 1.0 mm^3
Taipei Veteran General Hospital	133	70.7±9.1 [43-92]	47/86	GE Discovery MR750 (3T)	8-channel phased-array head coil	IR-FSPGR	TR/TE/TI = 9.4/4.0/450 msec; flip angle = 12°; NEX = 1; FOV = 256 x 256 mm^2; matrix size = 256 x 256; 172 axial slices; and voxel size = 1.0 x 1.0 x 1.0 mm^3
Tri-Service General Hospital	68	42.0±11.1 [21-62]	21/47	GE Discovery MR750 (3T)	8-channel phased-array head coil	BRAVO	TR/TE/TI = 10.17/4.16/450 msec; flip angle = 12°; NEX = 1; FOV = 256 × 256 mm^2; matrix size = 256 × 256; 172 axial slices; and voxel size = 1.0 x 1.0 x 1.0 mm^3
Kaohsiung Chang Gung Memorial Hospital	244	48.5±14.3 [20-75]	127/117	Siemens Skyra (3T)	20-channel phased-array head/neck coil	MPRAGE	TR/ TE/TI = 1800/2.7/900 msec; flip angle = 9°; NEX = 1; FOV = 230 x 230 mm^2; matrix size = 192 x 192; 144 sagittal slices; and voxel size = 1.2 x 1.2 x 1.2 mm^3
				GE Signa Excite (3T)	8-channel phased-array head coil	IR-FSPGR	TR/ TE/ TI = 9.5/3.9/450 msec; flip angle = 15°; NEX = 1; FOV = 240 × 240 mm^2; matrix size = 512 × 512; 110 axial slices; and voxel

							size = $0.47 \times 0.47 \times 1.3$ mm^3
Keelung Chang Gung Memorial Hospital	69	65.0±5.1 [52-77]	32/37	Siemens Skyra (3T)	20-channel phased-array head/neck coil	MPRAGE	TR/TE/TI = 2,200/2.45/900 msec; flip angle = 8°, NEX=1; FOV = 256 x 256 mm^2; matrix size = 256 x 256; 176 sagittal slices; and voxel size = 1.0 x 1.0 x 1.0 mm^3

TR, Repetition time; TE, echo time; TI, inversion time; NEX, number of excitations; FOV, field of view;

Supplementary Table 2. ROIs with significant BAG change in migraine patients compared with healthy controls

ROI index	Nearby Anatomical Location	BAG			t-value	FDR corrected p-value	Effect size (η_p^2)
		Healthy controls	Migraine	Difference			
56	Left Juxtapositional Lobule Cortex	-25.28 ± 28.42	-0.72 ± 24.39	24.56	5.08	<0.001	0.130
32	Left Planum Polare	-7.73 ± 12.15	3.27 ± 13.43	11.00	4.46	0.003	0.103
142	Left Middle Frontal Gyrus	-3.17 ± 17.09	8.45 ± 15.50	11.62	4.08	0.007	0.088
349	Right Middle Frontal Gyrus	1.83 ± 18.20	14.65 ± 15.67	12.82	4.03	0.007	0.086
88	Left Precentral Gyrus	-8.82 ± 20.72	4.97 ± 18.20	13.79	4.01	0.007	0.085
65	Left Superior Frontal Gyrus	-1.43 ± 30.92	15.90 ± 26.99	17.33	3.99	0.007	0.084
110	Left Paracingulate Gyrus	-15.10 ± 14.71	-2.35 ± 16.71	12.75	3.90	0.009	0.081
268	Right Precentral Gyrus	-28.96 ± 36.89	-3.93 ± 31.45	25.04	3.84	0.009	0.079
148	Left Superior Frontal Gyrus	-18.83 ± 16.37	-6.05 ± 17.68	12.77	3.77	0.011	0.076
332	Right Supramarginal Gyrus, posterior division	-0.42 ± 20.26	12.78 ± 17.98	13.20	3.69	0.013	0.073
55	Left Precentral Gyrus	-12.88 ± 20.77	1.30 ± 22.16	14.19	3.63	0.015	0.071
106	Left Frontal Pole	-9.12 ± 16.40	2.26 ± 16.05	11.38	3.59	0.015	0.069
183	Left Frontal Pole	-13.72 ± 21.92	0.16 ± 24.33	13.88	3.59	0.015	0.069
351	Right Middle Frontal Gyrus	2.48 ± 18.96	13.44 ± 15.59	10.95	3.44	0.023	0.064
390	Right Superior Frontal Gyrus	-1.32 ± 17.14	7.60 ± 16.76	8.92	3.42	0.023	0.063
48	Left Postcentral Gyrus	-10.41 ± 15.27	-0.24 ± 16.31	10.17	3.41	0.023	0.063
178	Left Paracingulate Gyrus	-12.16 ± 16.91	-2.38 ± 15.24	9.78	3.35	0.024	0.061
111	Left Precuneous Cortex	-4.37 ± 17.52	8.35 ± 18.85	12.71	3.34	0.024	0.061
109	Left Cingulate Gyrus, posterior division	-6.78 ± 13.70	3.16 ± 15.88	9.93	3.33	0.024	0.060
197	Left Cingulate Gyrus, posterior division	-8.10 ± 17.31	4.06 ± 18.50	12.17	3.31	0.024	0.060
365	Right Supramarginal Gyrus, posterior division	6.22 ± 17.21	17.89 ± 18.46	11.67	3.30	0.024	0.059
388	Right Middle Frontal Gyrus	-2.14 ± 15.64	8.07 ± 16.45	10.21	3.30	0.024	0.059
86	Left Precentral Gyrus	-1.98 ± 17.45	6.45 ± 15.57	8.43	3.27	0.024	0.058
345	Right Frontal Pole	4.99 ± 21.35	17.08 ± 18.69	12.09	3.26	0.024	0.058
108	Left Cingulate Gyrus, anterior division	-11.70 ± 13.65	-1.72 ± 15.08	9.98	3.24	0.025	0.057

382	Right Paracingulate Gyrus	-10.71 ± 12.79	-2.61 ± 15.14	8.10	3.16	0.031	0.054
310	Right Frontal Pole	0.55 ± 14.44	10.67 ± 17.45	10.12	3.15	0.031	0.054
189	Left Superior Frontal Gyrus	-11.54 ± 22.66	4.37 ± 25.74	15.91	3.14	0.031	0.054
113	Left Juxtapositional Lobule Cortex	-12.94 ± 29.68	4.12 ± 28.75	17.06	3.13	0.031	0.054
179	Left Frontal Pole	-9.91 ± 19.10	0.32 ± 16.90	10.23	3.10	0.032	0.053
160	Left Angular Gyrus	-1.43 ± 21.82	13.41 ± 23.05	14.85	3.10	0.032	0.053
61	Left Precentral Gyrus	-3.43 ± 22.56	8.50 ± 21.00	11.93	3.09	0.032	0.052
387	Right Superior Frontal Gyrus	-9.26 ± 20.74	-0.06 ± 19.85	9.20	3.07	0.033	0.052
137	Left Frontal Pole	-11.60 ± 18.69	1.77 ± 20.63	13.37	3.07	0.033	0.052
49	Left Juxtapositional Lobule Cortex	-9.68 ± 12.35	-0.46 ± 17.17	9.22	3.05	0.033	0.051
300	Right Supramarginal Gyrus, anterior division	3.88 ± 16.28	14.67 ± 18.81	10.79	3.04	0.033	0.051
85	Left Superior Parietal Lobule	-5.59 ± 40.36	18.71 ± 46.25	24.31	3.03	0.033	0.050
343	Right Frontal Pole	-4.17 ± 16.77	6.11 ± 16.98	10.28	3.02	0.033	0.050
391	Right Superior Frontal Gyrus	-6.82 ± 21.07	5.20 ± 25.48	12.02	3.01	0.033	0.050
333	Right Supramarginal Gyrus, posterior division	1.26 ± 18.14	12.23 ± 20.49	10.97	3.01	0.033	0.050
360	Right Paracingulate Gyrus	-10.96 ± 13.33	-2.43 ± 13.68	8.53	3.00	0.033	0.049
140	Left Middle Frontal Gyrus	-3.53 ± 16.11	6.15 ± 15.73	9.68	3.00	0.033	0.049
348	Right Frontal Pole	-3.80 ± 15.36	5.32 ± 15.88	9.12	2.99	0.033	0.049
181	Left Superior Frontal Gyrus	-4.37 ± 18.03	4.52 ± 15.03	8.90	2.95	0.036	0.048
54	Left Postcentral Gyrus	-8.14 ± 18.54	3.23 ± 20.33	11.36	2.95	0.036	0.048
33	Left Planum Temporale	-5.41 ± 14.27	4.06 ± 16.68	9.47	2.94	0.036	0.048
164	Left Lateral Occipital Cortex, superior division	2.19 ± 23.37	15.09 ± 23.10	12.90	2.91	0.037	0.047
267	Right Postcentral Gyrus	-6.49 ± 31.97	12.56 ± 31.34	19.06	2.91	0.037	0.047
266	Right Superior Frontal Gyrus	2.11 ± 24.03	16.59 ± 28.03	14.48	2.90	0.037	0.046
321	Right Frontal Pole	-6.15 ± 12.58	-0.45 ± 15.68	5.70	2.90	0.037	0.046
383	Right Frontal Pole	-5.49 ± 19.00	5.50 ± 20.42	10.99	2.90	0.037	0.046
182	Left Middle Frontal Gyrus	-4.43 ± 19.27	5.15 ± 18.21	9.58	2.89	0.037	0.046
287	Right Precuneous Cortex	1.41 ± 24.95	16.26 ± 23.95	14.85	2.88	0.037	0.046
153	Left Temporal Pole	-2.44 ± 12.55	5.28 ± 14.30	7.72	2.88	0.037	0.046

173	Left Frontal Pole	-15.82 ± 18.94	-2.80 ± 20.96	13.02	2.87	0.037	0.045
107	Left Cingulate Gyrus, anterior division	-11.30 ± 10.97	-3.82 ± 12.91	7.47	2.87	0.037	0.045
296	Right Supramarginal Gyrus, posterior division	8.82 ± 20.42	20.91 ± 18.96	12.10	2.86	0.037	0.045
44	Left Postcentral Gyrus	-9.55 ± 18.29	0.21 ± 16.39	9.76	2.83	0.040	0.044
364	Right Angular Gyrus	8.83 ± 17.76	19.11 ± 18.42	10.27	2.82	0.040	0.044
174	Left Paracingulate Gyrus	-15.34 ± 14.16	-6.62 ± 15.12	8.72	2.79	0.043	0.043
177	Left Cingulate Gyrus, anterior division	-12.31 ± 10.88	-5.36 ± 13.67	6.95	2.79	0.043	0.043
342	Right Frontal Pole	-1.57 ± 19.11	8.69 ± 18.83	10.26	2.78	0.043	0.043
395	Right Cingulate Gyrus, posterior division	-3.02 ± 17.50	6.16 ± 16.13	9.17	2.77	0.044	0.042
317	Right Juxtapositional Lobule Cortex	-15.34 ± 25.77	0.17 ± 26.49	15.51	2.76	0.044	0.042
406	Left Amygdala	-5.88 ± 11.44	0.59 ± 13.26	6.47	2.73	0.047	0.041
129	Left Angular Gyrus	0.65 ± 19.12	11.70 ± 22.65	11.05	2.71	0.050	0.041

ROI= region of interest, BAG= brain age gap; FDR= false discovery rate

Supplementary Table 3. The brain regions significantly associated with the clinical profile CV

ROI index	Nearby Anatomical Location	Correlation Coefficient	p-value
32	Left Planum Polare	0.311	0.007
54	Left Postcentral Gyrus	0.300	0.009
86	Left Precentral Gyrus	-0.254	0.028
111	Left Precuneous Cortex	0.265	0.021
129	Left Angular Gyrus	0.284	0.014
160	Left Angular Gyrus	0.254	0.028
164	Left Lateral Occipital Cortex, superior division	0.286	0.013
183	Left Frontal Pole	0.248	0.032
364	Right Angular Gyrus	0.242	0.036
365	Right Supramarginal Gyrus, posterior division	0.266	0.021
387	Right Superior Frontal Gyrus	0.247	0.032

CV= canonical variate; ROI= region of interest

Supplementary Figure 1.

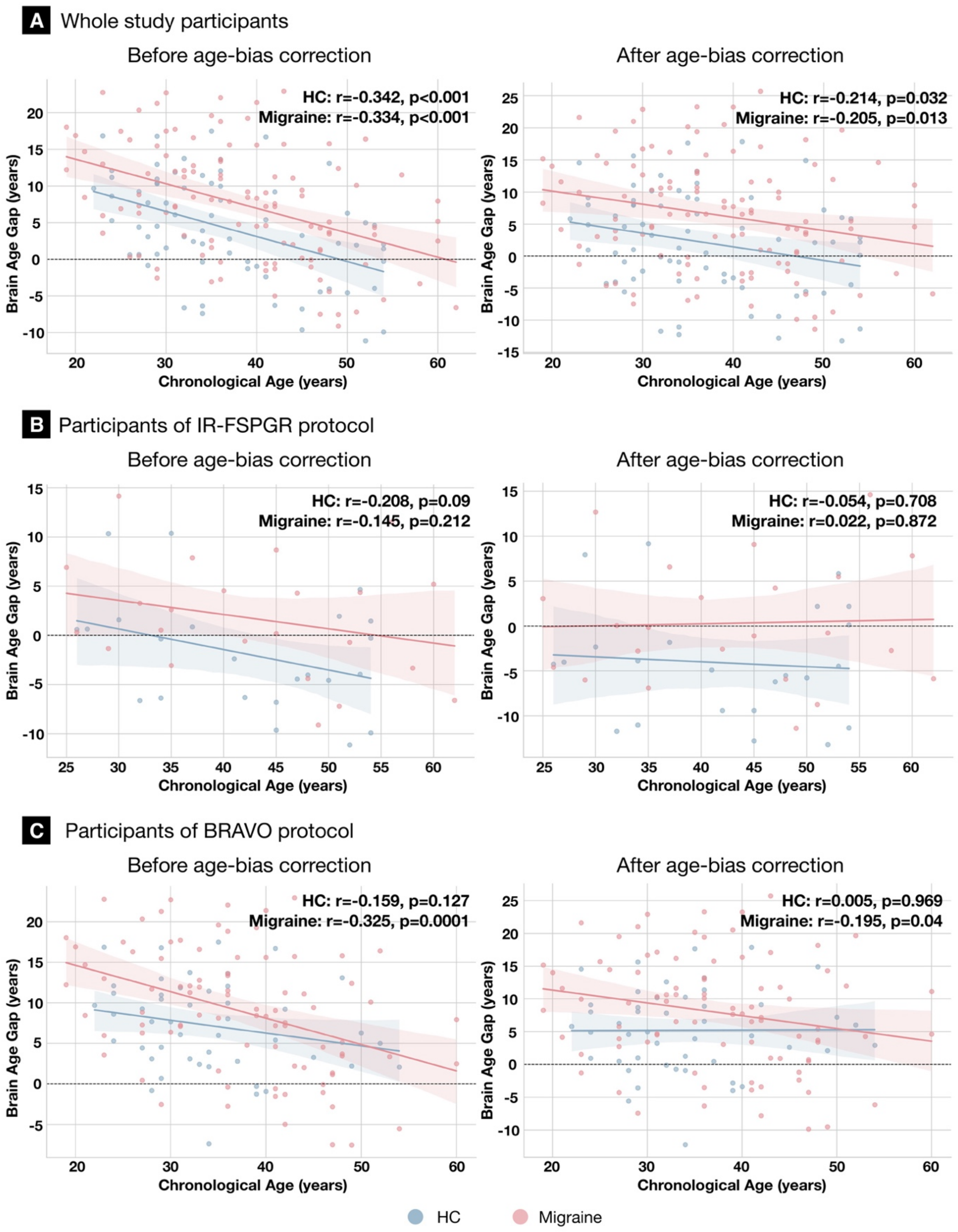

Supplementary Figure 1. Evaluation of correction effectiveness. (A) Before correction, Pearson's correlation analysis showed significant negative correlations

between BAG and chronological age in both groups (HC: $r = -0.342$, 95% CI: [-0.5052, -0.1790], $p < 0.001$; Migraine: $r = -0.334$, 95% CI: [-0.4696, -0.1993], $p < 0.001$). After correction, these correlations were reduced (HC: $r = -0.214$, 95% CI: [-0.4090, -0.0194], $p = 0.032$; Migraine: $r = -0.205$, 95% CI: [-0.3664, -0.0435], $p = 0.013$), although some residual bias persisted. This is expected, as brain age estimation is known to be sensitive to training data properties, age distribution, and dataset heterogeneity. (B, C) Protocol-stratified analyses further confirmed correction effectiveness. For IR-FSPGR (n = 46), correlations decreased from HC: $r = -0.208$ (95% CI: [-0.4578, 0.0410], $p = 0.09$), Migraine: $r = -0.145$ (95% CI: [-0.3779, 0.0888], $p = 0.212$) to HC: $r = -0.054$ (95% CI: [-0.3523, 0.2435], $p = 0.708$), Migraine: $r = 0.022$ (95% CI: [-0.2570, 0.3006], $p = 0.872$). For BRAVO (n = 134), correlations decreased from HC: $r = -0.159$ (95% CI: [-0.3644, 0.0470], $p = 0.127$), Migraine: $r = -0.325$ (95% CI: [-0.4851, -0.1663], $p = 0.0001$) to HC: $r = 0.005$ (95% CI: [-0.2410, 0.2503], $p = 0.969$), Migraine: $r = -0.195$ (95% CI: [-0.3850, -0.0041], $p = 0.04$).

BAG = brain age gap; BRAVO = inversion-recovery-prepared fast spoiled gradient recalled brain volume; HC = healthy controls; IR-FSPGR = inversion recovery-prepared fast spoiled gradient-recalled.
